# Supplementary figures and images for: Predicting mean ribosome load for 5’UTR of any length using deep learning
Source: PLoS Comput Biol. 2021 May 10;17(5):e1008982. doi: 10.1371/journal.pcbi.1008982 (PMC8136849; doi:10.1371/journal.pcbi.1008982)

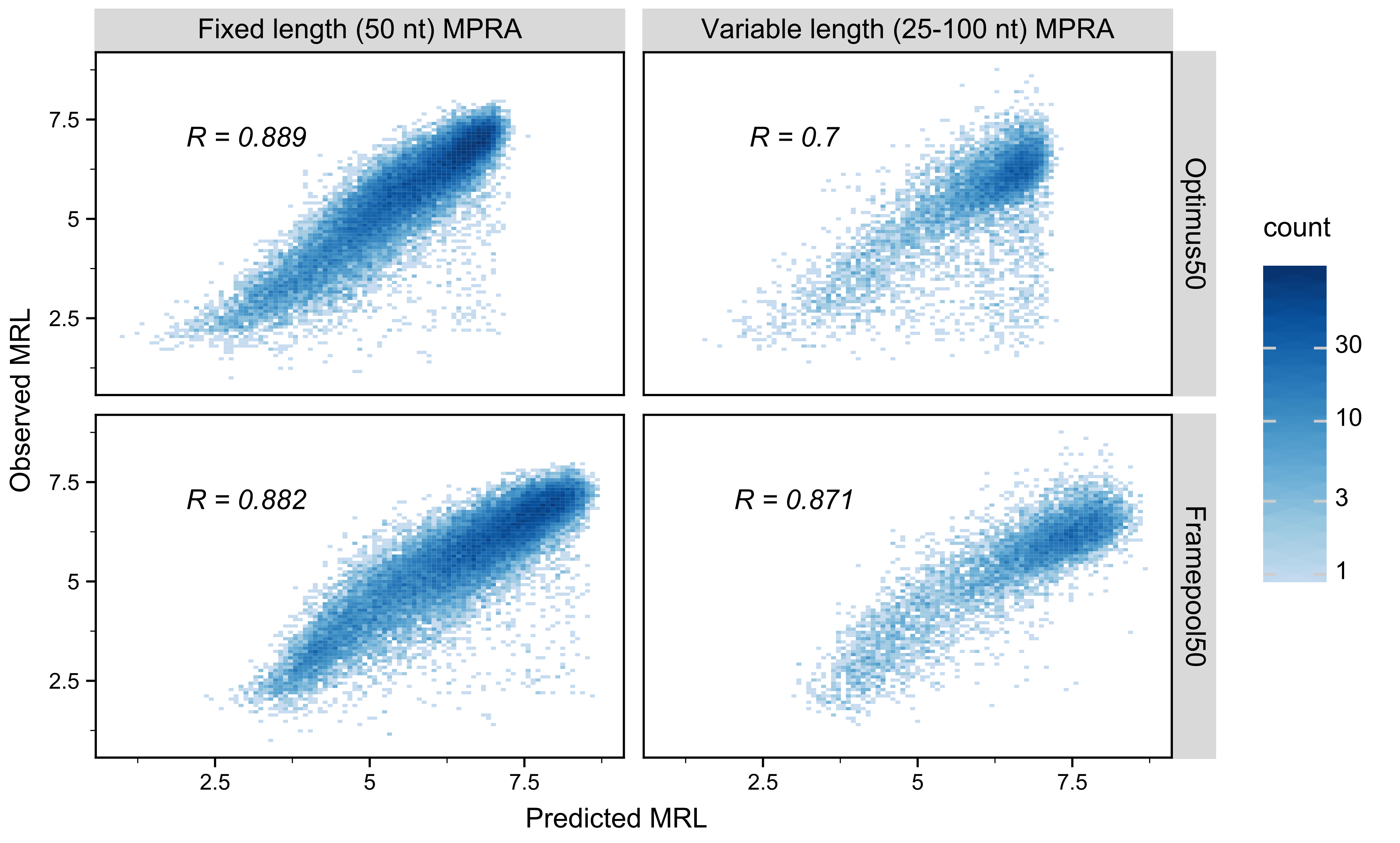

Supplement: S1 Fig — On 50 nt human sequences, the two models perform equivalently, but FramePool50 generalizes much better to longer sequences. (TIF) [file pcbi.1008982.s001.tif]

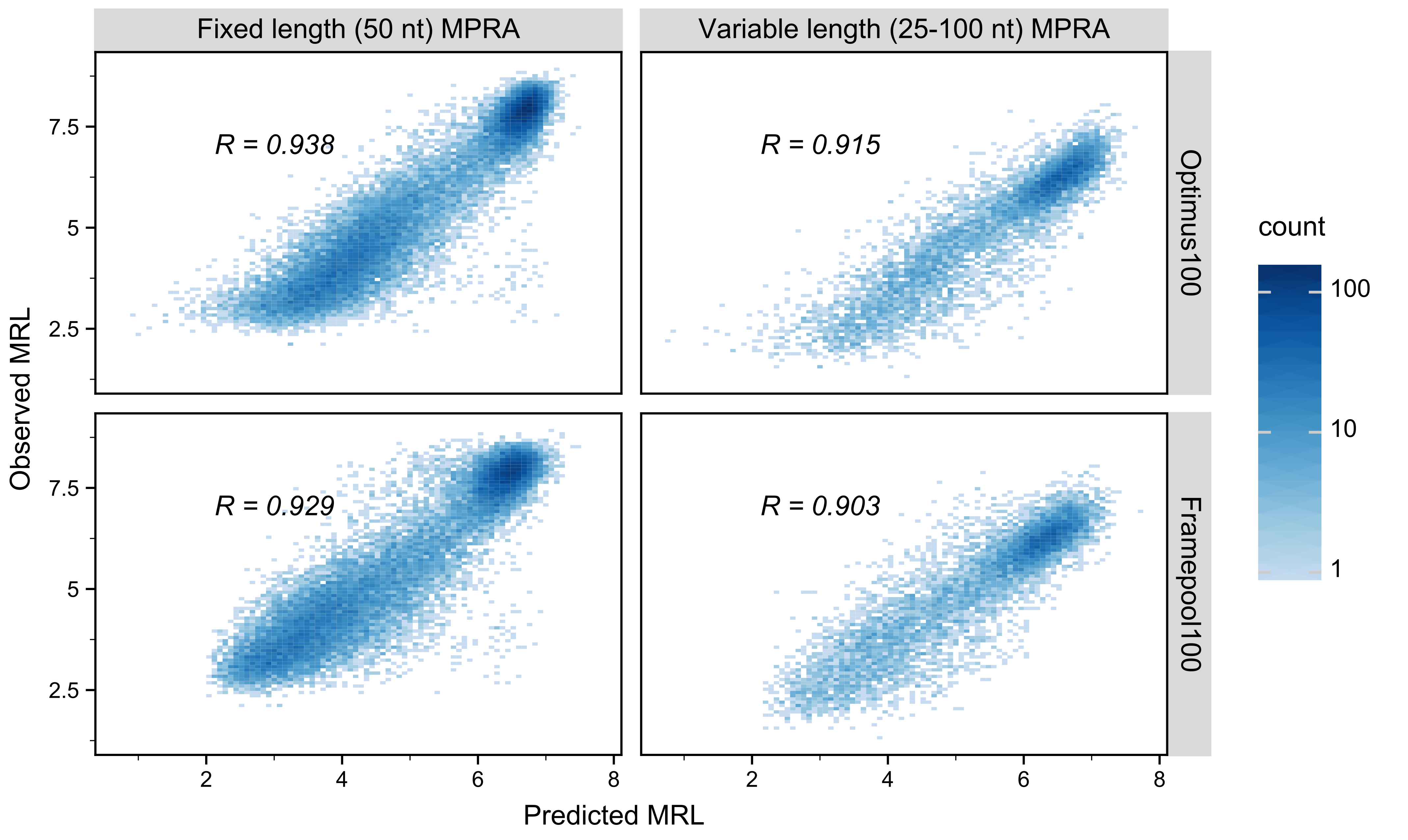

Supplement: S2 Fig — (TIF) [file pcbi.1008982.s002.tif]

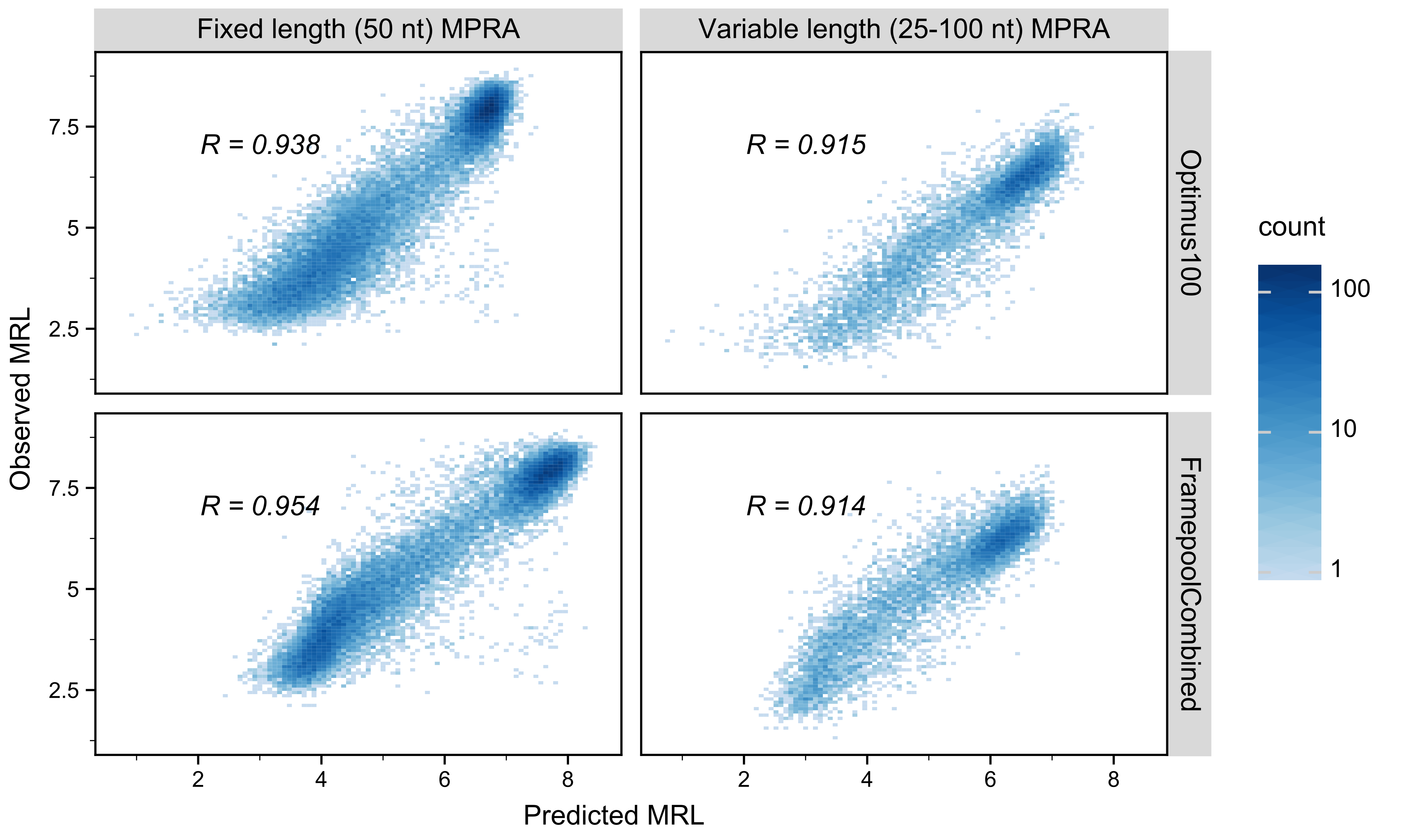

Supplement: S3 Fig — (TIF) [file pcbi.1008982.s003.tif]

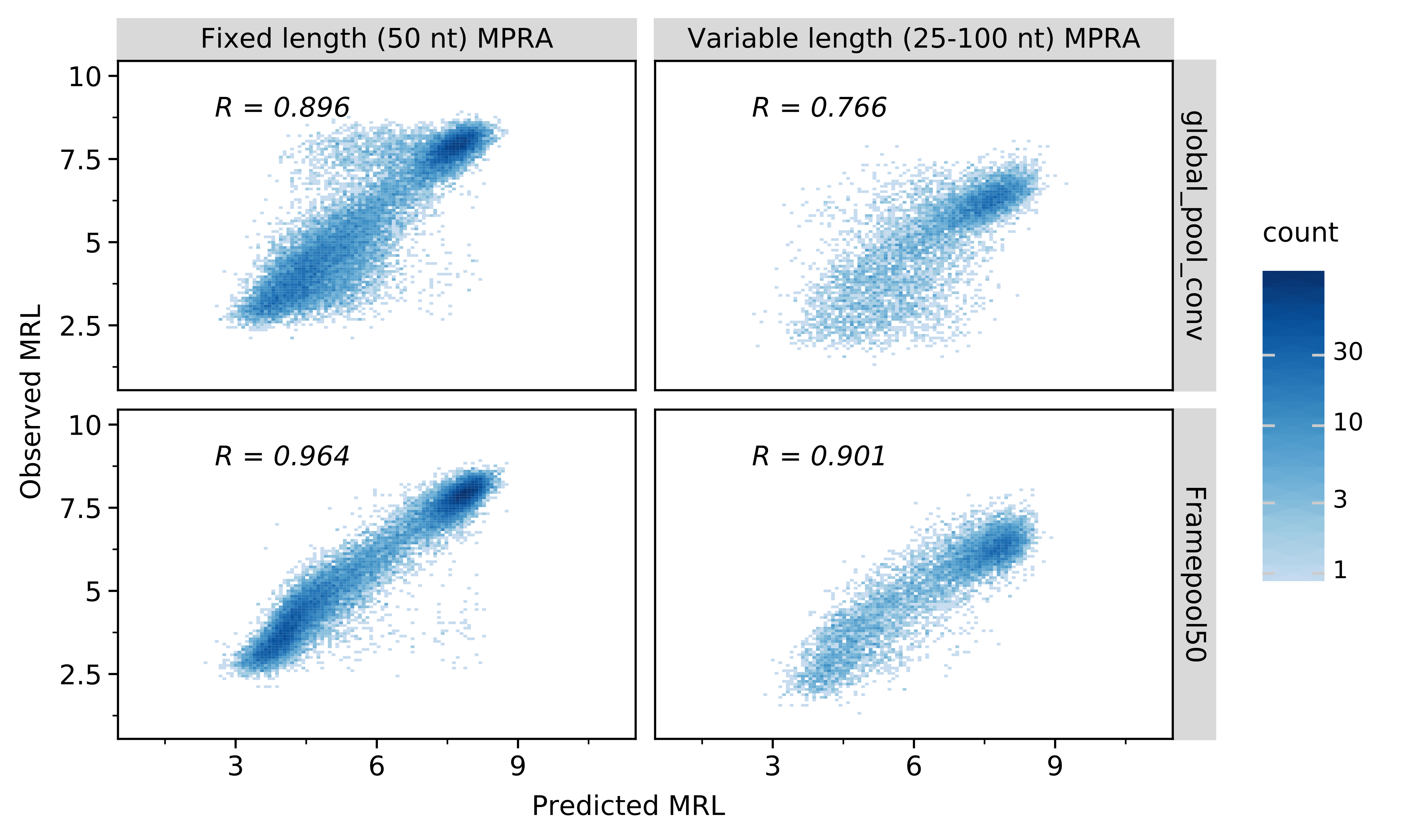

Supplement: S4 Fig — The global_pool_conv uses the same hyperparameters as FramePool50, but frame pooling is replaced with standard global max and average pooling. The global_pool_conv, which has no knowledge of frame, performs worse than FramePool50. (TIF) [file pcbi.1008982.s004.tif]

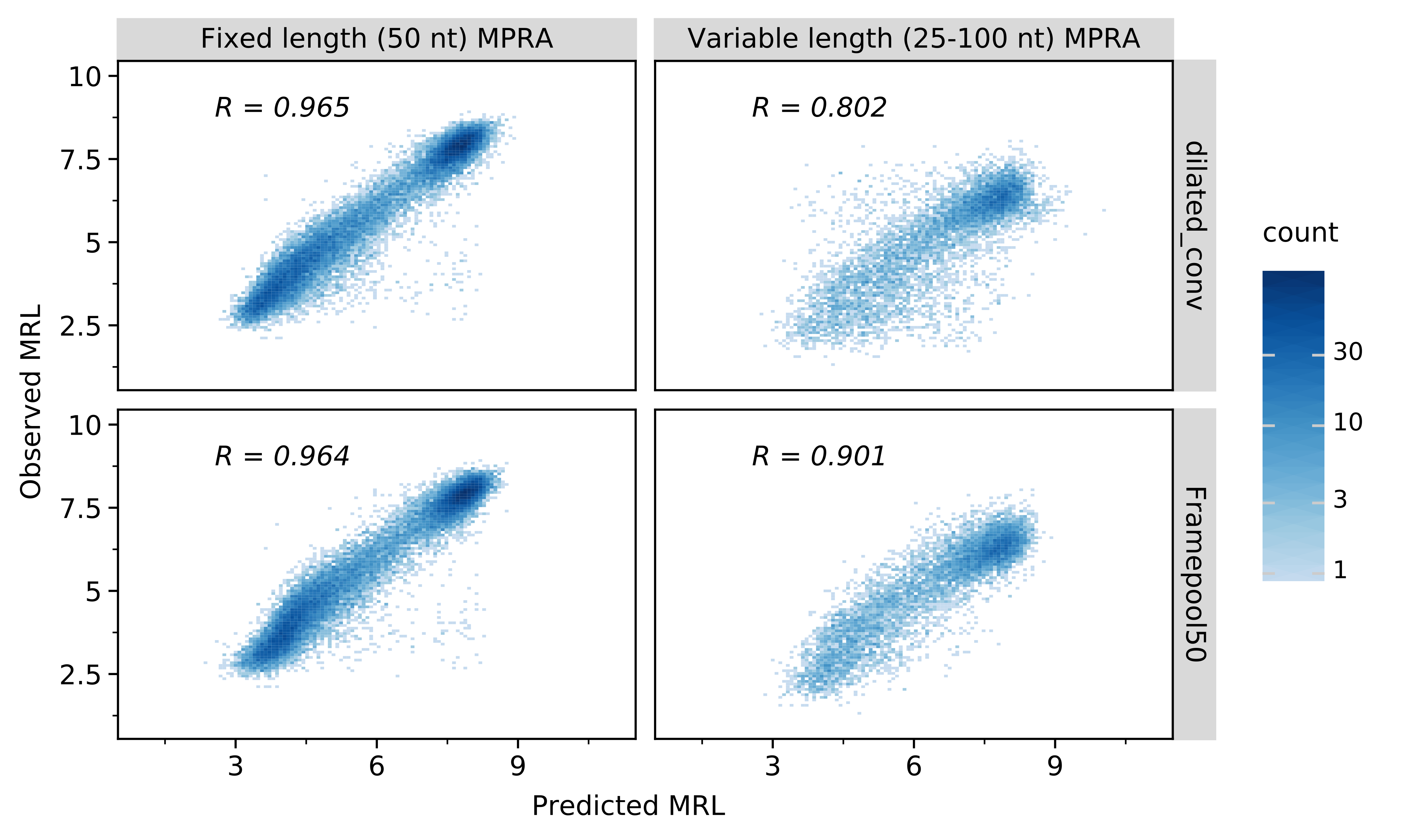

Supplement: S5 Fig — The dilated_conv uses the same hyperparameters as FramePool50, but frame pooling is replaced with standard global max and average pooling and, additionally, dilations are used to expand the receptive field size to 43. On fixed length data, the large receptive field size likely allows the dilated_conv model to effectively infer the frame, but this fails to fully generalize to the variable length sequences. As a result, FramePool50 generalizes markedly better to the variable length sequences. (TIF) [file pcbi.1008982.s005.tif]

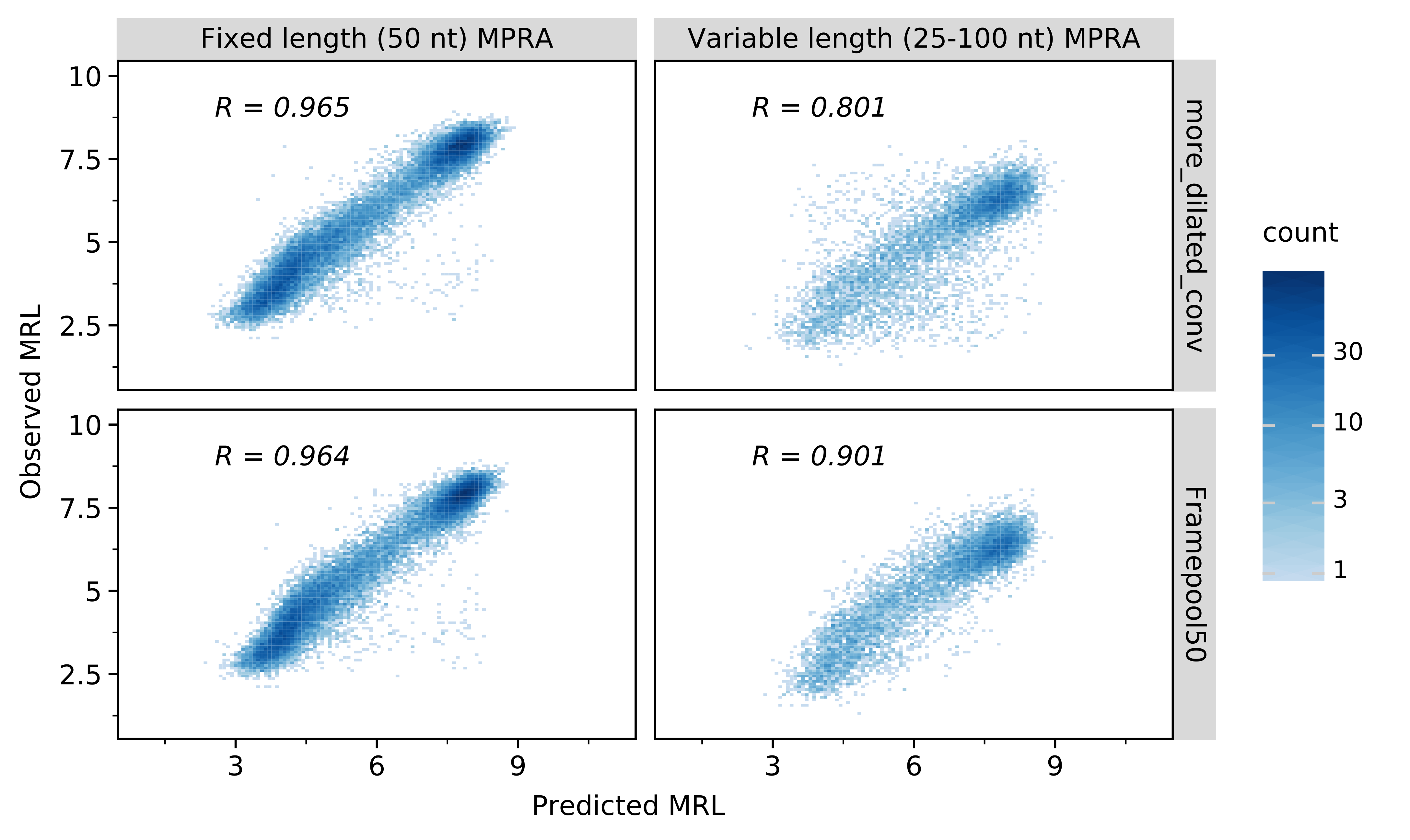

Supplement: S6 Fig — The more_dilated_conv model is similar to the dilated_conv model, but has one additional dilated convolutional layer (with a dilation factor of 8). The additional dilation brings no improvement. (TIF) [file pcbi.1008982.s006.tif]

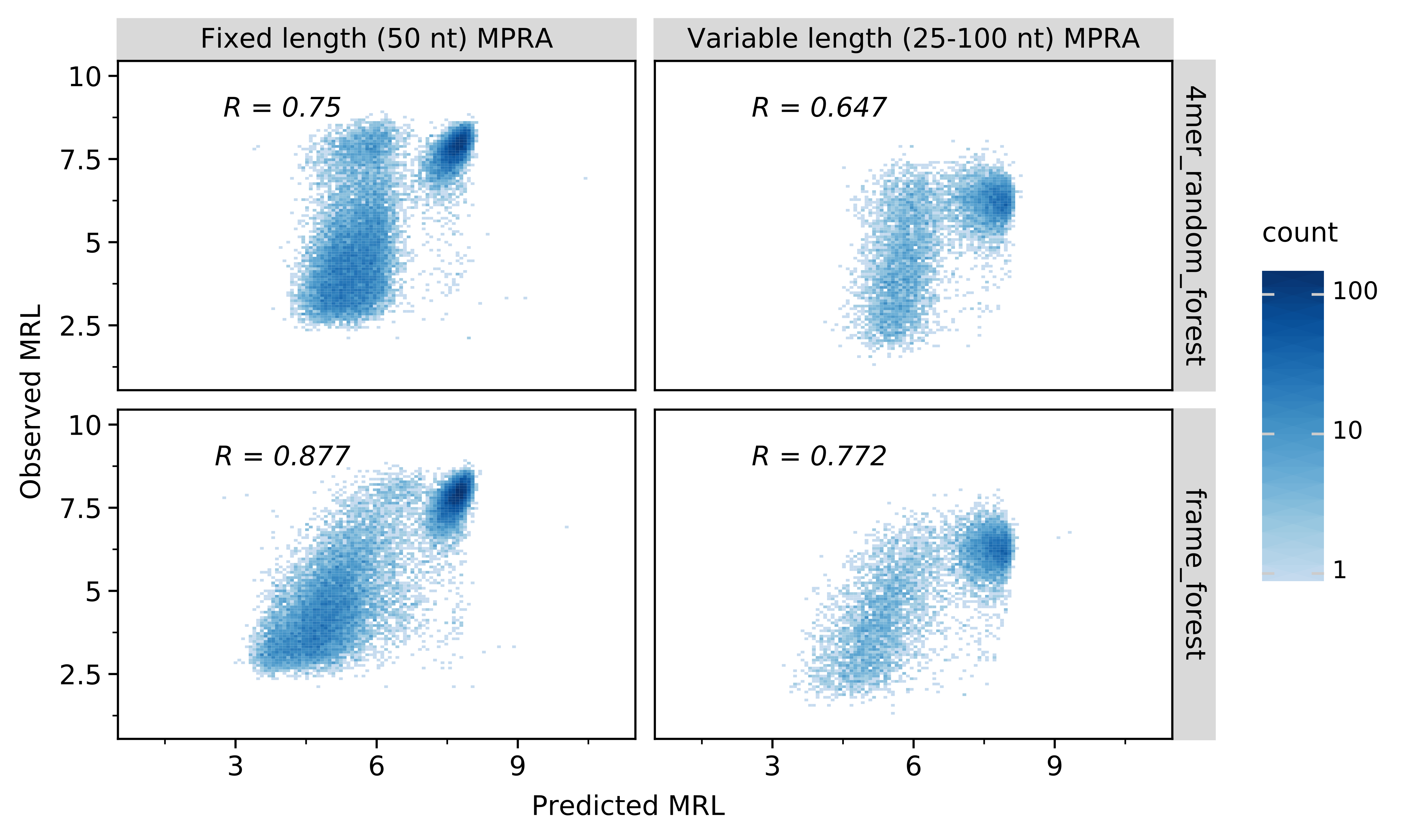

Supplement: S7 Fig — The 4mer_random_forest gets as input a 4^4-dimensional vector of 4-mer counts, which offers no frame information. The frame_forest gets as input a 3*(4^4)-dimensional vector of 4-mer counts for each frame. The frame information allows the frame_forest model to markedly outperform the frame-unaware random forest. (TIF) [file pcbi.1008982.s007.tif]

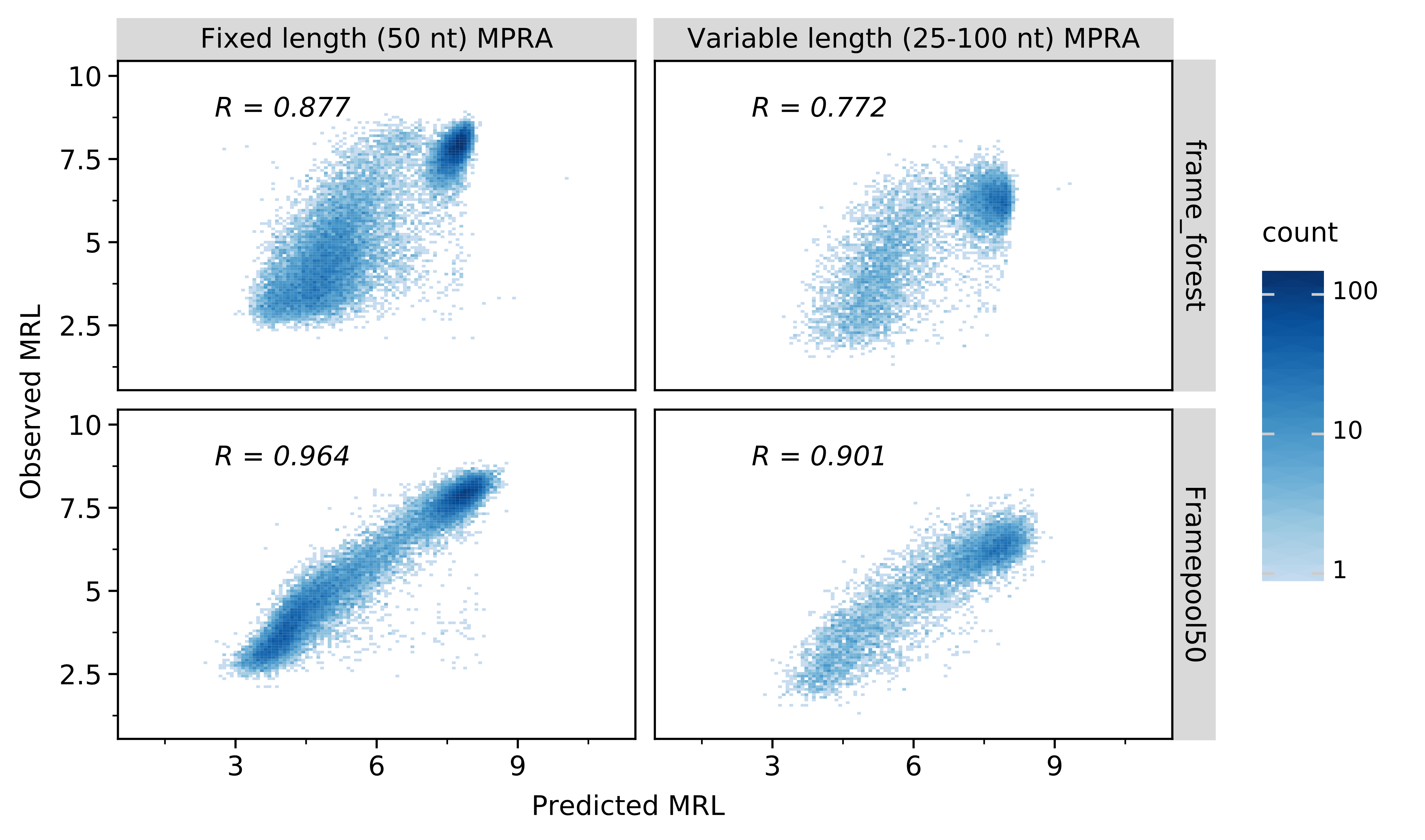

Supplement: S8 Fig — FramePool50 offers better performance than the random forest based model. (TIF) [file pcbi.1008982.s008.tif]

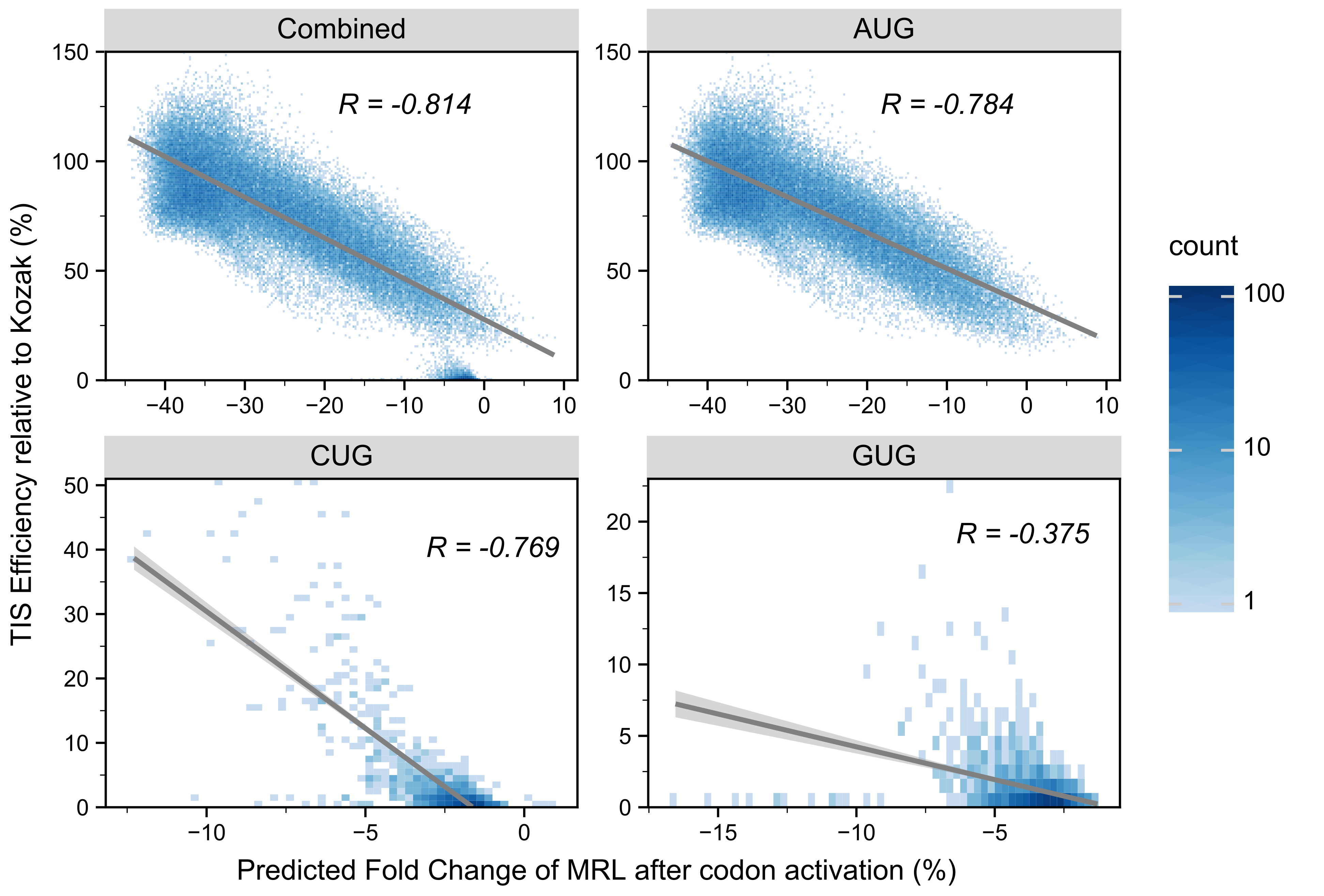

Supplement: S9 Fig — (TIF) [file pcbi.1008982.s009.tif]

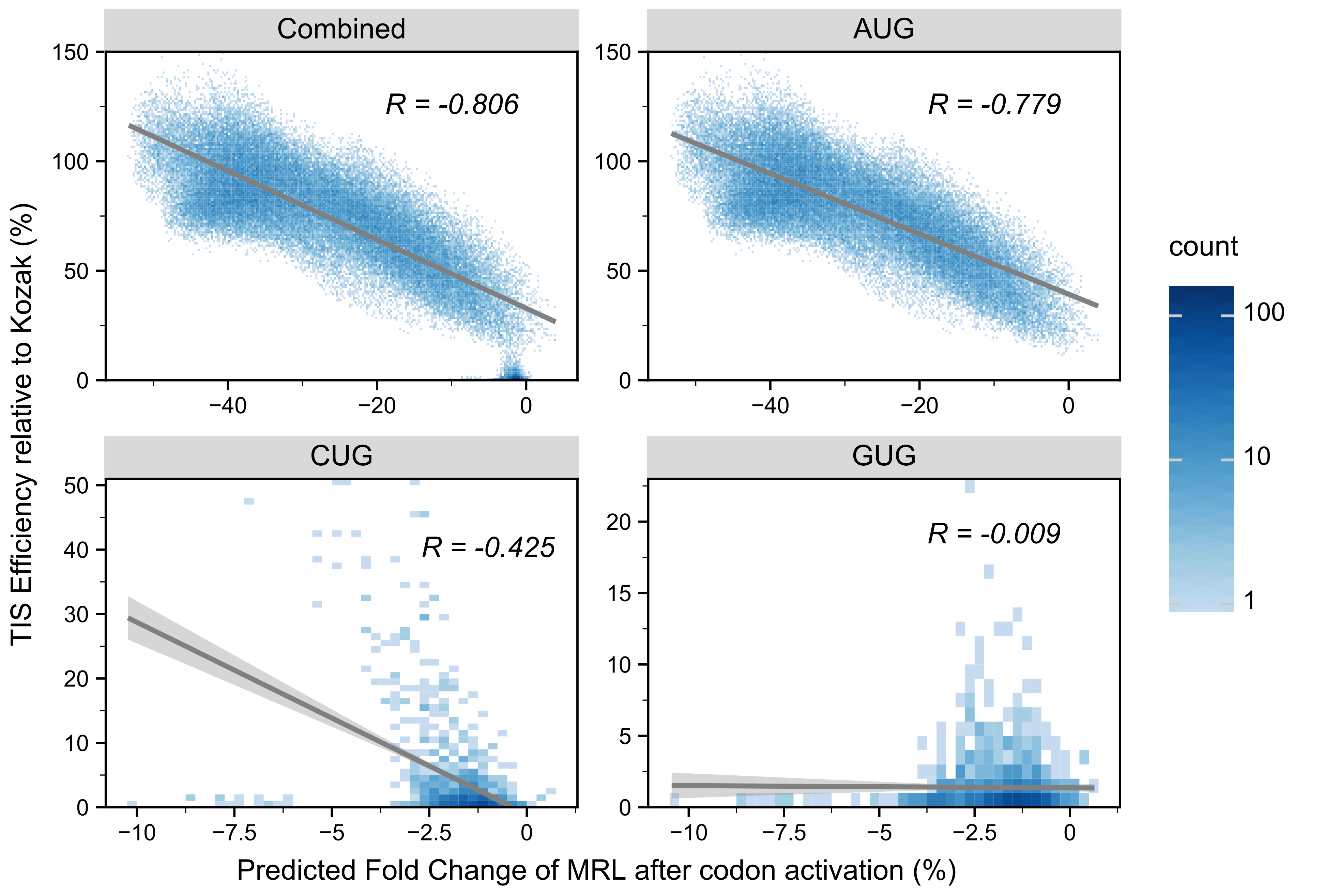

Supplement: S10 Fig — (TIF) [file pcbi.1008982.s010.tif]

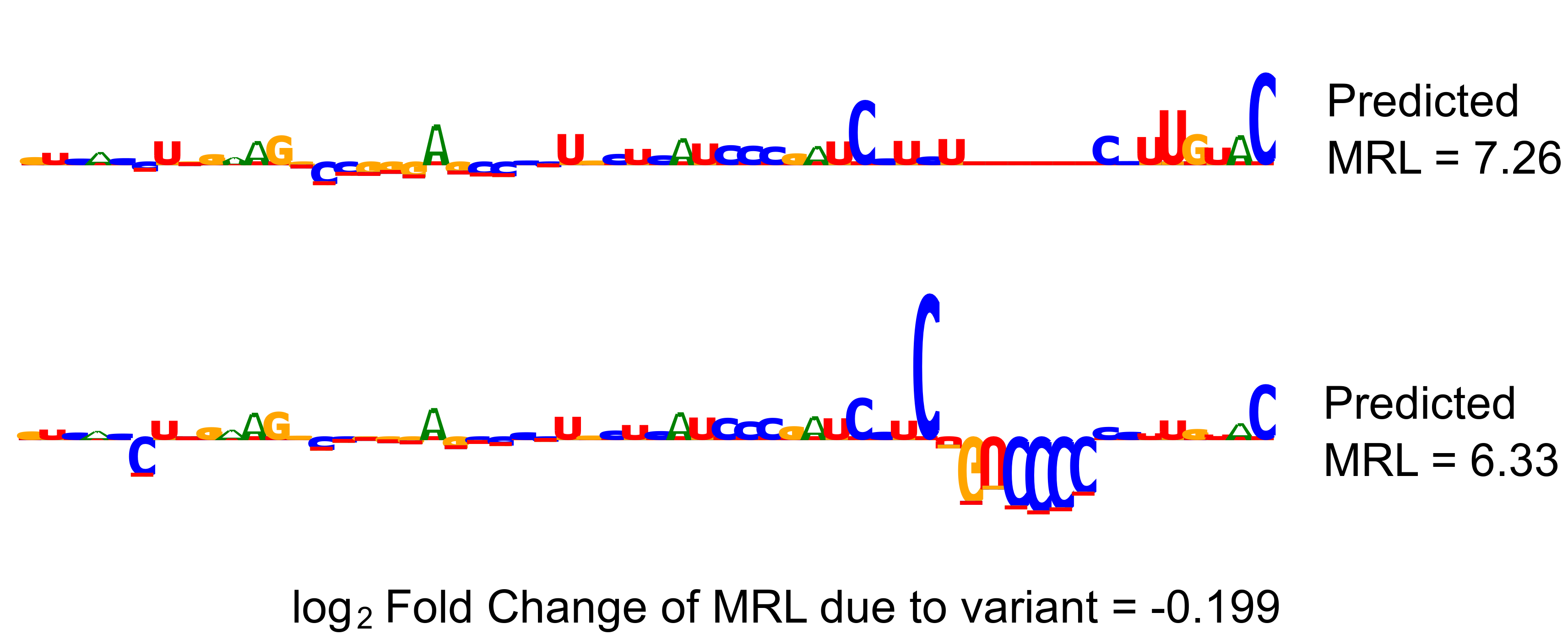

Supplement: S11 Fig — Contribution logos are aligned for visualization purposes. Since a frame pooling model can predict MRL for sequences of any length, it can in principle quantify the effect of any indel or complex variant. This is done by predicting MRL for both the wildtype and the mutated sequence and then computing the log-fold change in MRL. In this case, the insertion of the GUCCCC motif is predicted to depress MRL. (TIF) [file pcbi.1008982.s011.tif]

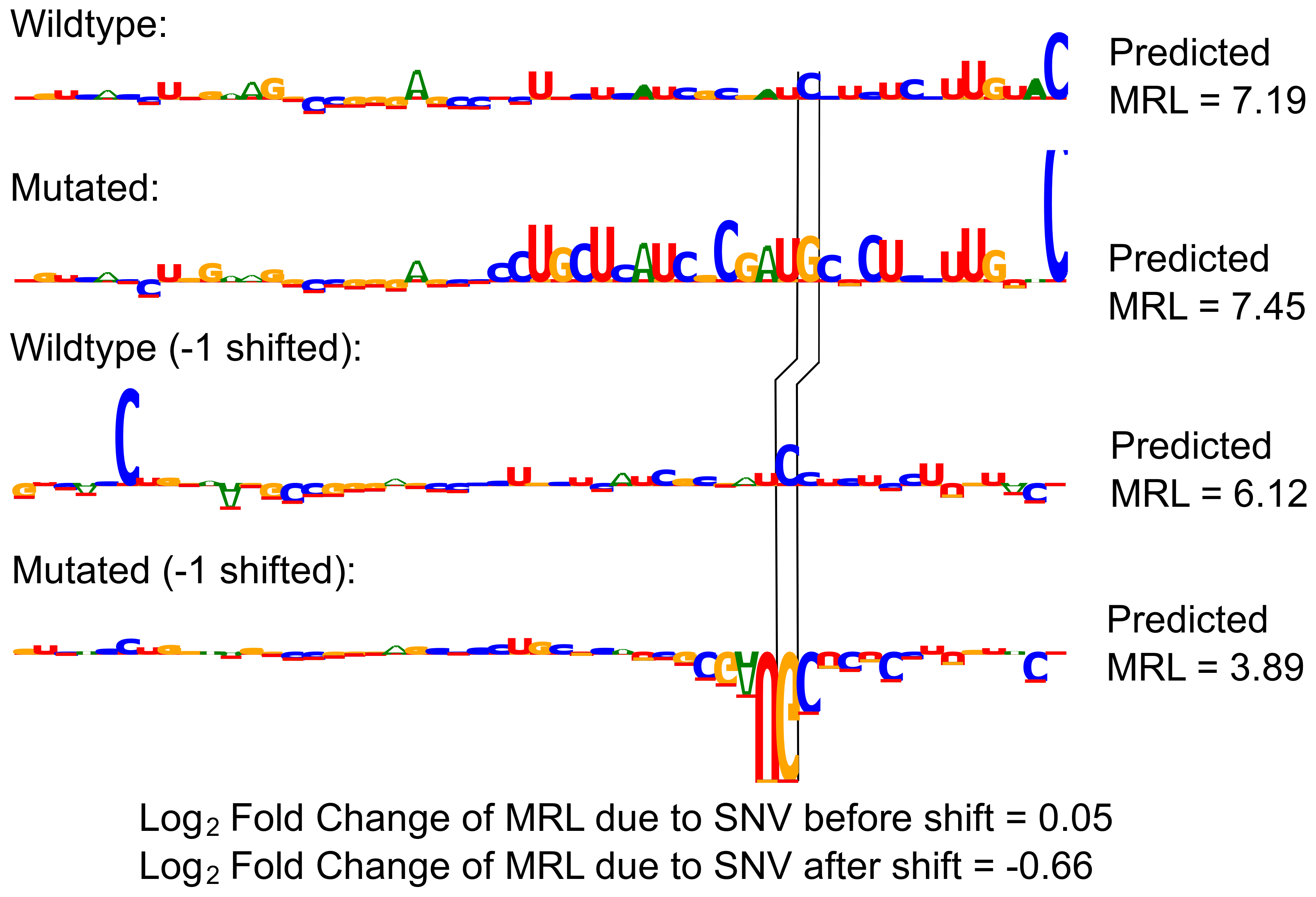

Supplement: S12 Fig — Because the new uTIS is in-frame with respect to the canonical start codon, it has only a small effect on the MRL predicted by the model. However, such a variant could be lengthening the canonical protein. To alert the user to such in-frame uTIS creation or deletion events, the kipoi version of the model also reports the effect of the variant after shifting the frame of reference. This shift “tricks” the model into treating an in-frame variant as out-of-frame. If the variant effect on MRL after such a shift is strongly negative, whereas the original effect is small, it is an indication that a variant may be lengthening the canonical protein. (TIF) [file pcbi.1008982.s012.tif]

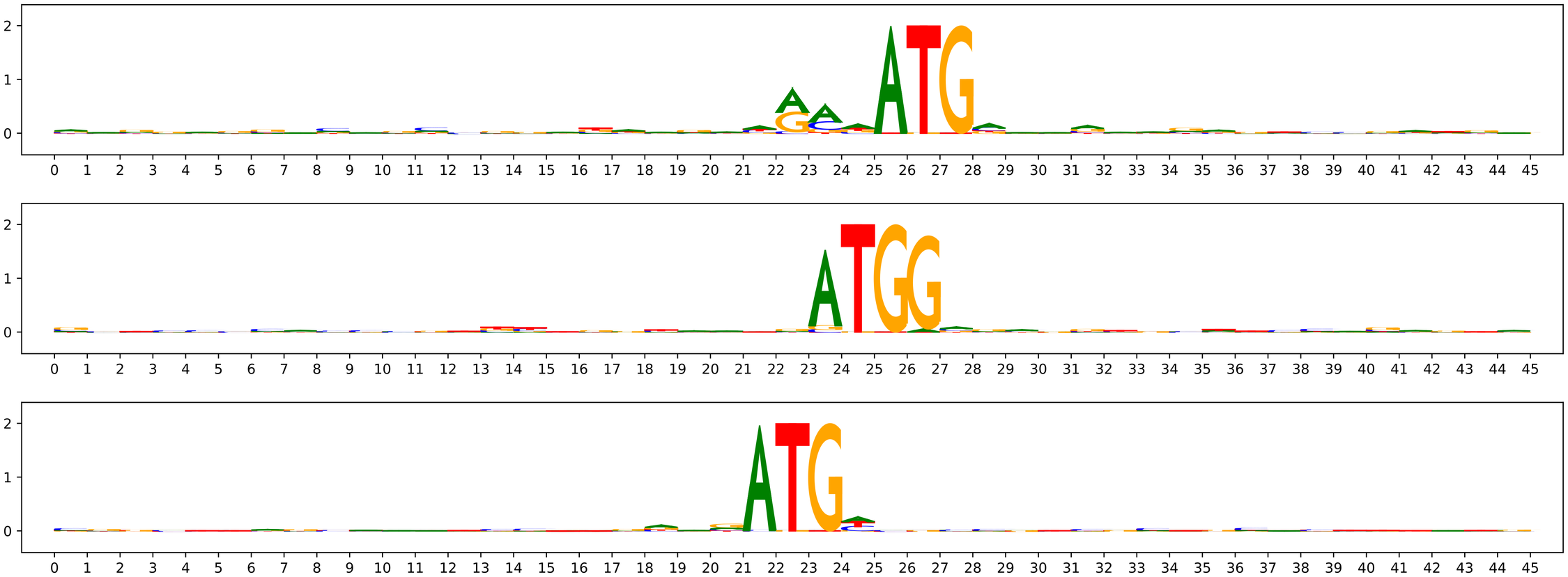

Supplement: S13 Fig — (TIF) [file pcbi.1008982.s013.tif]

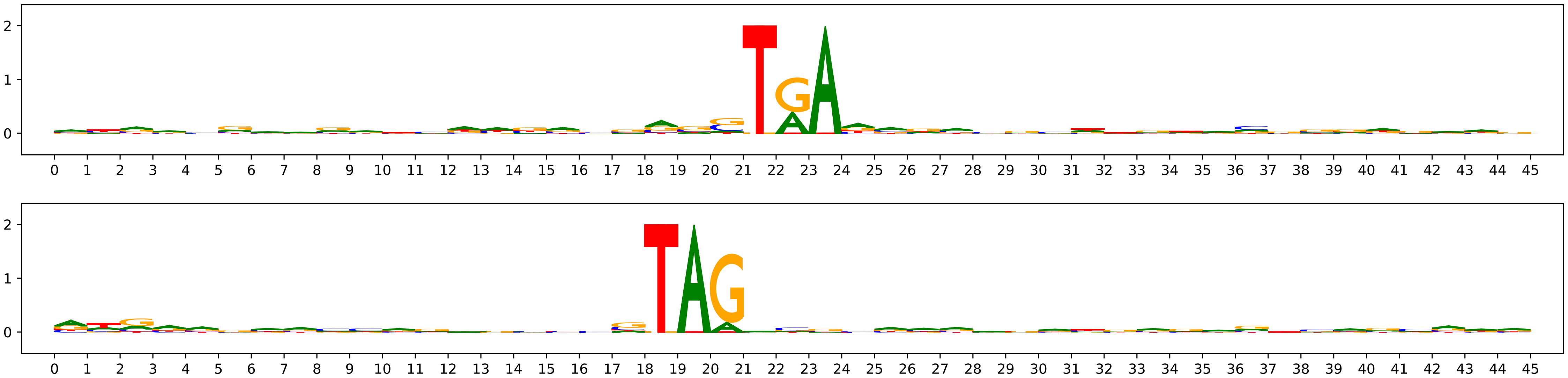

Supplement: S14 Fig — (TIF) [file pcbi.1008982.s014.tif]

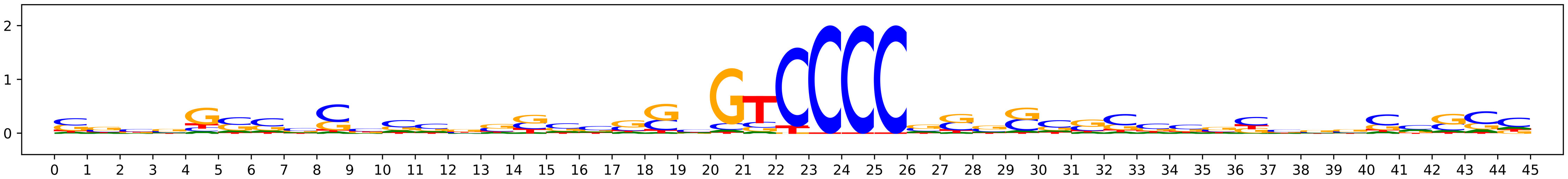

Supplement: S15 Fig — (TIF) [file pcbi.1008982.s015.tif]

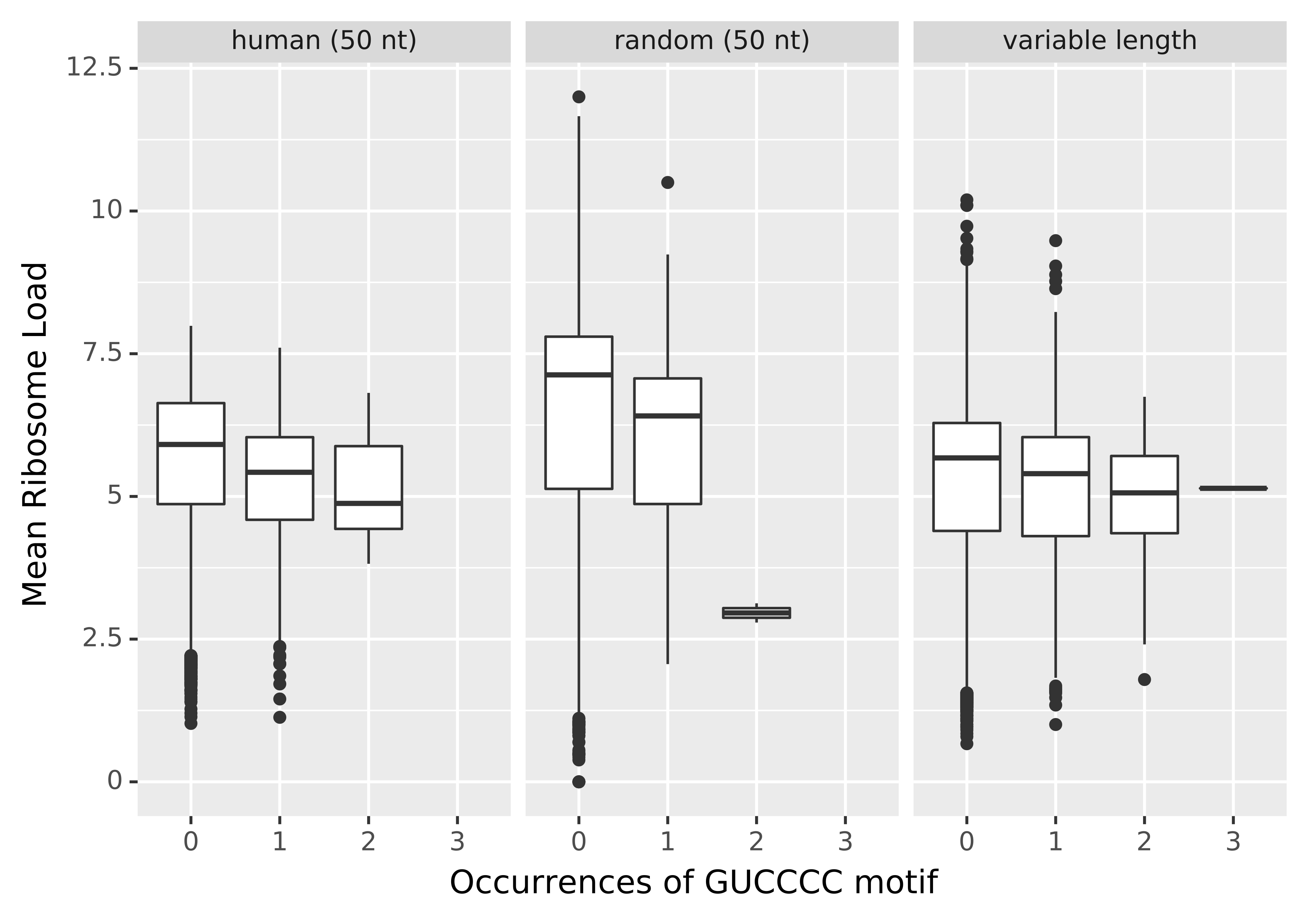

Supplement: S16 Fig — In each dataset, presence of the GUCCCC motif leads to a repression of mean ribosome load compared to when the motif is not present. (TIF) [file pcbi.1008982.s016.tif]

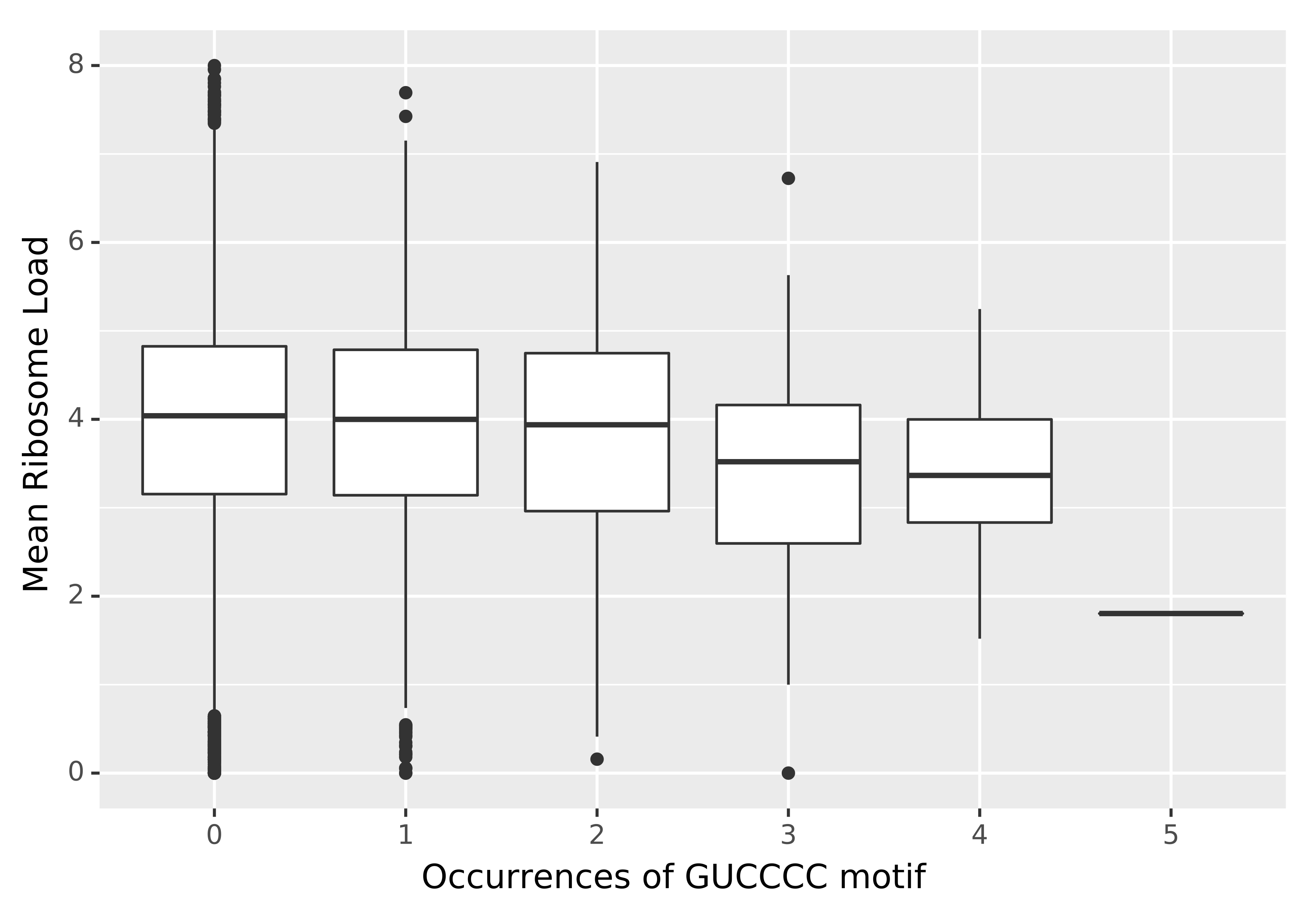

Supplement: S17 Fig — (TIF) [file pcbi.1008982.s017.tif]

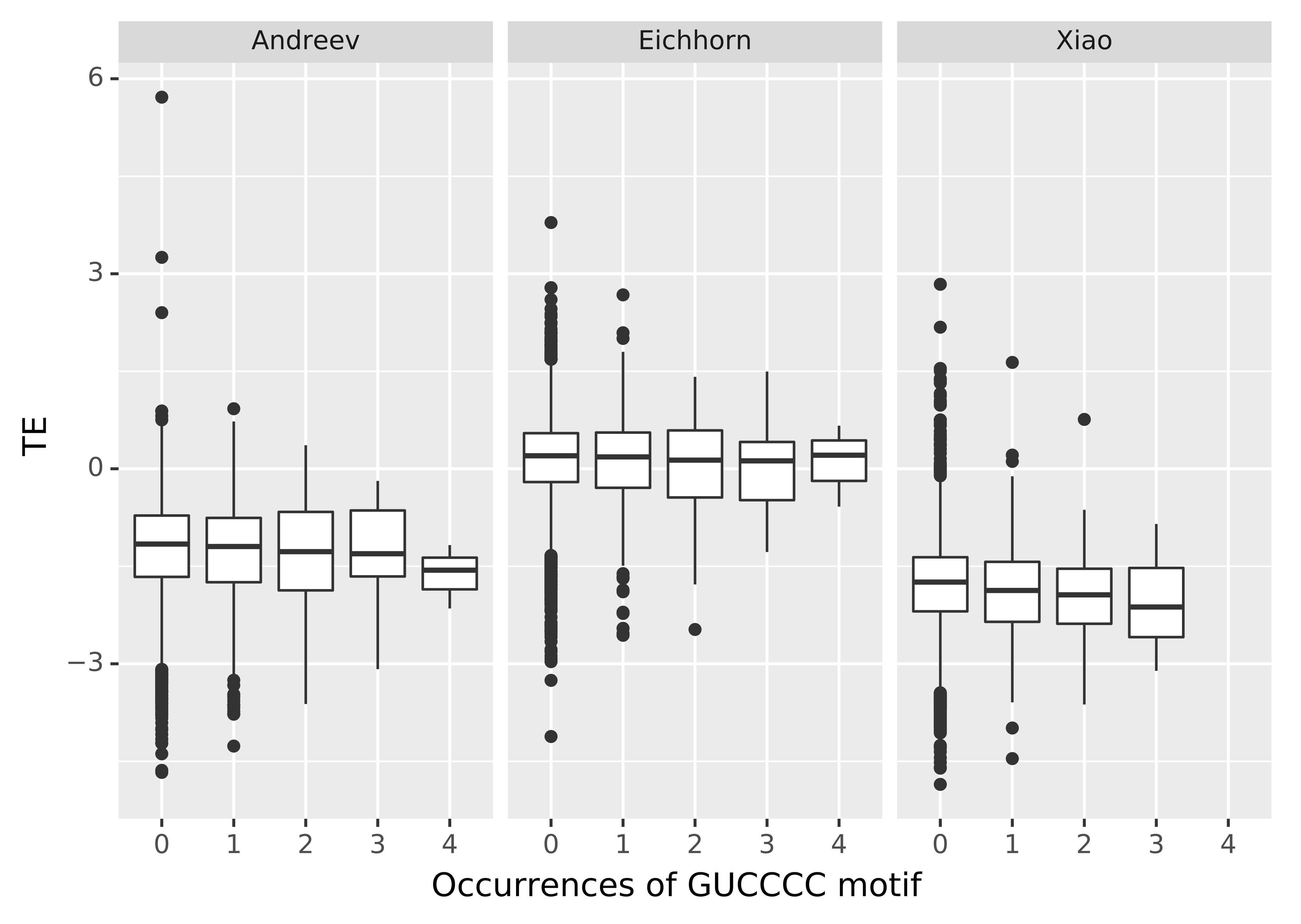

Supplement: S18 Fig — TE is a measure of translation efficiency given by the log-ratio of ribo-seq reads to rna-seq reads covering a specific gene. (TIF) [file pcbi.1008982.s018.tif]

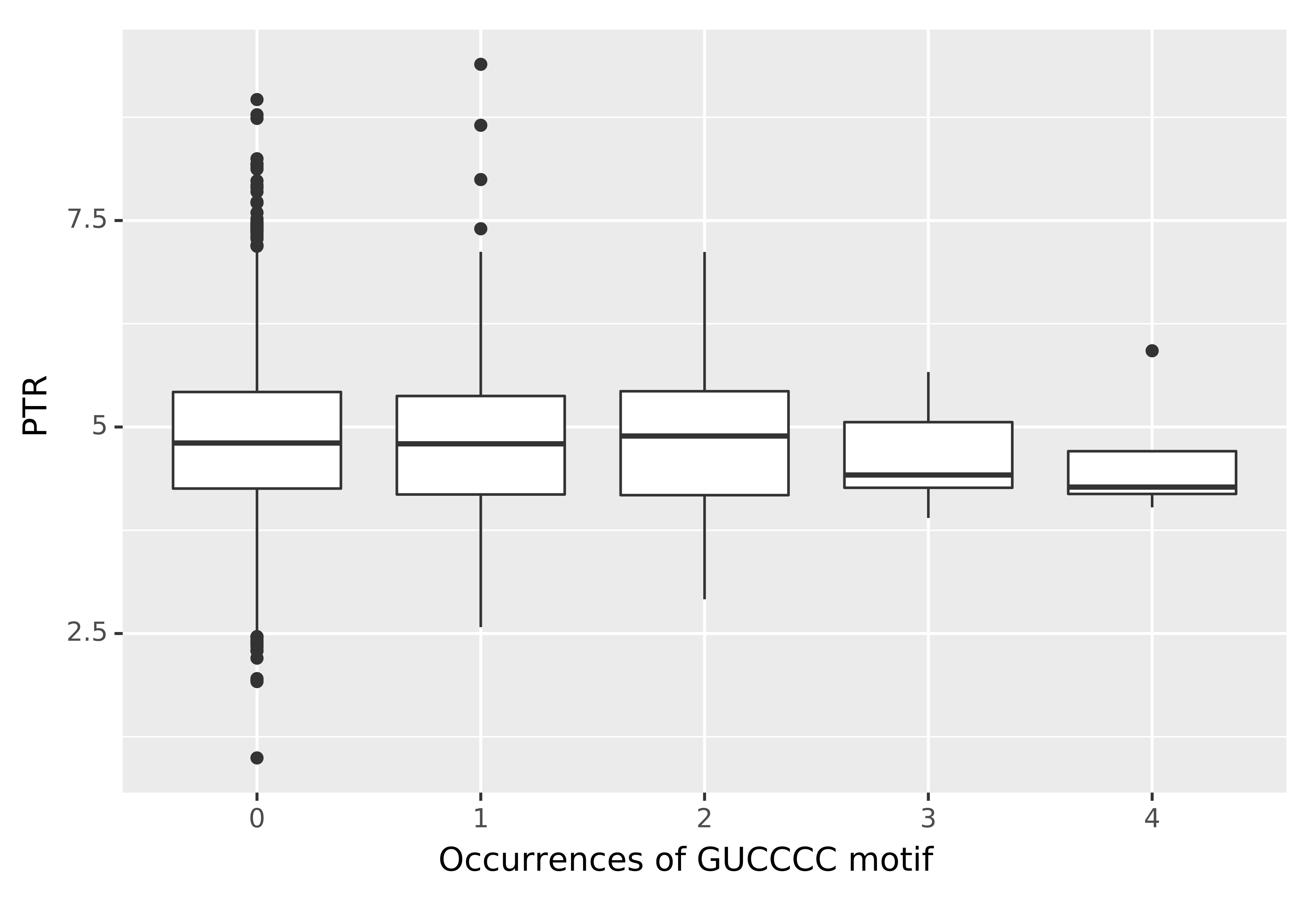

Supplement: S19 Fig — PTR is the protein-to-RNA ratio. (TIF) [file pcbi.1008982.s019.tif]

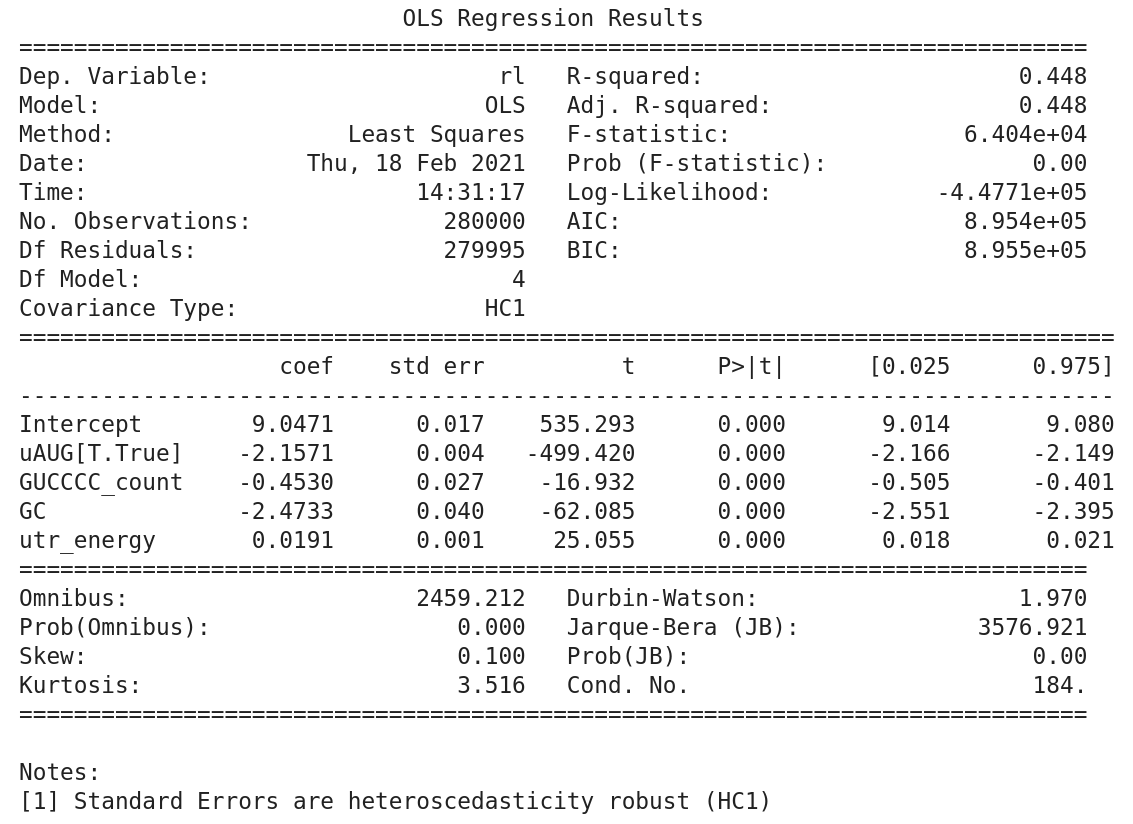

Supplement: S5 Table — We see that the GUCCCC motif has a statistically significant effect on MRL even when controlling for GC content and (predicted) UTR min folding energy. (PNG) [file pcbi.1008982.s024.png]
