# Supplementary material for: Predicting mean ribosome load for 5’UTR of any length using deep learning
Source: PLoS Comput Biol. 2021 May 10;17(5):e1008982. doi: 10.1371/journal.pcbi.1008982 (PMC8136849; doi:10.1371/journal.pcbi.1008982)
Supplement: Attachment — Submitted filename: Response to Review.pdf [file pcbi.1008982.s027.pdf]

Dear Dr Radivojac, dear Dr Noble,

We are submitting the revised version of our manuscript entitled “Predicting Mean Ribosome Load for 5’UTR of any length using Deep Learning”. The revision addresses all points raised by the reviewers with the following changes:

1. We added supplementary figures to show the performance of FramePool100 and FramePoolCombined on MPRA data (Fig S2, S3).
2. We compared our frame pooling approach to conventional global pooling strategies which also generalize to arbitrary length sequences. We show that frame pooling offers considerably better generalization performance to variable length sequences than these alternatives (Fig S4-6)
3. We compared our frame pooling approach to random forest models trained on 3-mer and 4-mer counts. We show that our approach outperforms the random forest models, even if the random forests are also supplied with frame information (Fig S7, S8).
4. We repeated the uTIS strength prediction analysis for FramePool50 and FramePool100. We find generally similar results, with the exception that FramePool100 does worse on non-AUG codons, likely due to its smaller training set (Fig S9, S10).
5. We added additional text and supplementary figures to explain how the model scores indels (Fig S11) and to explain the procedure of shifting the frame to detect variants which may be lengthening the canonical coding sequence (Fig S12).
6. We performed model interpretation using Tf-Modisco (Shrikumar et al. 2018). The motifs found by Tf-Modisco correspond to known features of translational regulation (Fig S13, S14) and additionally include one, to our knowledge, novel motif (GUCCCC Fig S15). The motif significantly associated with repression of MRL in all MPRA datasets (Fig S16), but there was no clear association between the motif and translational repression in endogenous data (Fig S17-19, Table S6)

Moreover, we are submitting a point-by-point response to the reviewers as well as the revised manuscript and a version with highlighted changes as requested.

We thank the reviewers and the editorial boards for their helpful comments which led to a substantially improved manuscript.

We are very much looking forward to your decision.

On behalf of all authors,

Julien Gagneur

Reviewer #1:

Review of Karollus, Avsec, Gangeur.

The authors seek to improve on the Optimus algorithm developed by George Seelig's lab for predicting functional characteristics and repercussions of 5' untranslated region variants. These earlier algorithms have length limitations, and as the authors describe, the majority of human 5'UTRs are longer than the 50 to 100 nt cutoff. Additionally, positional weight in this algorithm was a concern. By pooling by reading frame contexts, the authors have addressed this concern, as well as improved the ability to predict on longer sequences, more characteristic of human 5'UTRs. Such a tool would be useful in the early detection of de novo variants that could lead to disease. The authors have incorporated this tool into the Kipio API for use with standard variant call format (VCF).

Section Summaries and Notes

Section 1: The authors describe the frame pooling approach and explain its importance in not generating positional weights when training models. Three models are generated, one trained on the 50 nt dataset, one on the 25-100 variable nt dataset, and a combination of the two.

Section 2: The FramePool50 model Is tested on MPRA data and is shown to better predict than Optimus50 for sequences it was not trained on. It is not surprising that a model that was not trained on the experimental data performed poorly (Optimus50), but still, it is impressive that the authors' model performed well on a dataset it was not trained on.

Note, typo on line 209, the truncated data tests are said to be shown in Fig S2, but this data is shown in Fig S1.

Response: The typo has been fixed.

R1: Also, apologies if I missed it, but how does the Optimus100 model perform compared to the Framepool100 model when trained and tested on the variable data? I would assume they are similar. How about the combined model? Some additional supplemental figures would be nice here.

Response: We have added two new supplementary figures showing the performance of FramePool100 compared to Optimus100 on fixed and variable-length data, and the performance of FramePoolCombined (Fig S2-3). The performance is very similar, although Optimus100 has a small edge compared to FramePool100 (Pearson correlations of 0.915 vs. 0.903 respectively).

R1: Section 3: One of the FramePool models accurately predicts strength of effects of upstream translation initiation sites (mutating AGG to AUG). As expected, correlations are negative as strong uTIS's decrease MRL comparatively more than weak ones. The model also predicts, although not as well, these effects in the context of alternate start codons CUG and GUG.

Why did the authors choose the FramePoolCombined for this task? Is it more comprehensive as it is a combination of the two models?

Response: Yes, FramePoolCombined was chosen as it has seen a larger variety of sequences during its training than the other models. Nevertheless, we have repeated the analysis also for FramePool50 and FramePool100 (Fig S9-10). The results are generally similar, except that FramePool100 does considerably worse on non-AUG uTIS. This is most likely due to its smaller training set.

R1: Section 4: The FramePool model predictions on endogenous data are correlated with actual measurements from that data. A slight, but significant, correlation is seen here. Agree with the authors' point that endogenous data is the result of other contributing sequences and processes than just the 5'UTR here, which is likely the reason the correlation wasn't stronger. Predictions perform slightly better than the Optimus' ones for the datasets.

Section 5: In silico mutation of nucleotides throughout the 5'UTR. Positions expected to impact highly tend to be more evolutionarily conserved.

Section 6: Workflow described, weight matrix used to display effect size and direction for 5'UTR positions along an example gene.

Major Questions to Address:

1) I was confused in section 6 around lines 388-390, how to interpret the results of the "shifting". I understand the concept, that an in frame uTIS will just lengthen the protein, and therefore the MRL may not change much, but am confused about how this is presented by the analysis, or how to interpret results. Please expand upon this.

Response: We have added more explanations at the end of the paragraph. We have additionally provided in the supplementary information a synthetic example sequence with an in-frame AUG creation (Fig S12). For that sequence, the predicted effect of the in-frame AUG creation is a small positive on MRL. After shifting the frame of reference, the effect is strongly negative, because the model is tricked into believing that the uTIS is created out of frame. In that way the user is alerted to a possible lengthening of the canonical protein.

R1: 2) How does the model deal with insertions/deletions?

Response: We have added a supplementary figure (Fig S11) which shows how the model can be used to estimate a variant effect for an indel. We make use of the fact that our model has no length restrictions, and thus can in principle predict MRL for any input sequence. Thus we can predict MRL for the sequence with and without the indel. The fold change in predicted MRL then gives the estimate for the effect of the indel.

R1: 3) Does framepooling, which will shift the apparent start codon, matter in terms of how far the variant is from this "newly created" start codon?

Response: The reviewer asks about effects of the distance between a variant and a "newly created" start codon. It is not entirely clear what is meant here. A variant creating an upstream AUG is necessarily located at the position of the upstream AUG. One may ask whether the distance between upstream out-of-frame AUG to the canonical AUG is captured

by the model. The model does not encode distance information. We hope this addresses the reviewer's question.

R1: Decision: Recommend publication with minor revisions to address concerns/questions, possibly additional supplemental figures fleshing out initial testing of models in section 2.

Reviewer #2:

The authors predict the mean ribosome load from 5'UTR sequences. Overall, the paper is well written, and has a sound methodology towards predicting the translation rate. The main contribution of the paper is the framepooling operation within a neural network where a convolutional neural network takes into account the three possible frames individually by taking the global average and max pooling from each of them. This allows the model to learn features from arbitrary length sequences by having full coverage of the sequences.

Pros:
1. The model performs better or at a similar level of the Optimus models. Specially it performs really well in terms of correlation score for longer than 100nt sequences.
2. The frame pooling operation is novel in the context of this problem.
3. The model performs fairly well in predicting strength of upstream transcription initiation sites.
4. The model achieves fairly well PhyloP conservation scores from its prediction of single nucleotide variants showing it may indeed be learning biologically relevant subsequences.
5. The authors tested their method on endogenous data as MPRA data might not account for the spuriousness that is experienced there. Even though the correlation scores are pretty low, I thank the authors for undertaking this part.
Cons:
1. The absence of a convolutional neural network that takes arbitrary length sequences and uses traditional dilated convolution or max pooling operations mean I am not really sure the frame pooling operation is indeed necessary.

Response: To address this concern, we have trained two additional models. One uses the same hyperparameters as the frame pool models, but does global pooling (max and average) rather than frame pooling. When trained on the fixed length data, it does considerably worse than FramePool50 on the fixed-length test-set, and furthermore generalizes poorly to the variable length data (Fig S4). This is likely due to the lack of frame information inherent to this model.

The second model uses dilated convolutions (it has three layers, as all other models, but the second is dilated with factor 2, the third with factor 4). The result is a receptive field size of 43. This much enlarged receptive field size likely enables the model to learn the frames in fixed-length 50nt data. However, the generalization performance to variable length data is once again worse than when using frame pooling (Fig S5). Adding yet another layer of dilation (factor 8) does not provide an additional performance boost (Fig S6).

R2: 2. No comparison with a random forest that just takes as input 3-grams or 4-grams. This solves the arbitrary length issue, and from experience, it's hard to do better than a random forest. So, it is essential that there is a comparison to gauge if a neural network is even necessary.

Response: To address this concern, we trained random forests that take as input the counts of all possible 3-mers and 4-mers in the sequence. These random forests perform well but cannot match the performance of FramePool50 (Fig S7). To see if this is a result of the lack of frame information, we next trained a "framed" forest (a random forest which gets as input the k-mer counts for each frame separately). This frame-aware random forest performs better than its unframed counterpart, once again underlining the predictive value of frame information, but nevertheless still performs worse than FramePool50 (Fig S8). We used a bayesian hyperparameter framework (hyperopt, (Bergstra, Yamins, and Cox 2013)) to optimize the random forest hyperparameters, but only achieved very small improvements compared to the default parameters in this way. Increasing the window size to 5-mers also yielded no further improvements.

Possibly, convolutional neural nets are more efficient than random forests in this use-case, due to their ability to easily learn PWM-like distributed representations of different sequence features.

R2: 3. One salient point of using a neural network might be to interpret features learnt by the convolutional filters. It will be interesting to see if they are learning motifs but there is not much material on this in the paper.

Response: To get a better picture of motifs the model is learning, we computed contribution scores for all 50nt sequences (those from the fixed length MPRA and those from the truncated human data). We then used Tf-Modisco (Shrikumar et al. 2018) to aggregate the contribution scores and find the most common motifs, so as to get a broad overview of what the model looks at. The motifs found correspond to known features of translational regulation (start codons, stop codons (likely designating small uORF) and Kozak-like motifs, Fig S13-14). Additionally Tf-Modisco found one, to our knowledge, novel motif candidate (GUCCCC, Fig S15).

The presence of this motif is associated with a repression of mean ribosome load in all MPRA datasets, including the truncated human sequences (Fig S16). This effect persists when controlling for GC content and UTR min folding energy, indicating that the GUCCCC motif is not just a proxy used by the model for GC content (Table S5). In the endogenous datasets, however, we do not see a clear effect associated with this motif (Fig S17-19). This could be because of confounding in the endogenous datasets.

R2: The paper does a good job of presenting the results in a clear way. The authors have also deposited their code and data properly which is always appreciated. I thank the authors for their hard work and would like to see their responses to my cons comments.

Response: We thank this and the other reviewer for the positive feedback and constructive suggestions.

Bergstra, James, Daniel Yamins, and David Cox. 2013. “Making a Science of Model Search: Hyperparameter Optimization in Hundreds of Dimensions for Vision Architectures.” In *Proceedings of the 30th International Conference on Machine Learning*, edited by Sanjoy Dasgupta and David McAllester, 28:115–23. Proceedings of Machine Learning Research. Atlanta, Georgia, USA: PMLR.

Shrikumar, Avanti, Katherine Tian, Anna Shcherbina, Žiga Avsec, Abhimanyu Banerjee, Mahfuza Sharmin, Surag Nair, and Anshul Kundaje. 2018. “Tf-Modisco v0. 4.4. 2-Alpha.” *arXiv Preprint arXiv:1811. 00416*.
