# Supplementary material for: Predicting mean ribosome load for 5’UTR of any length using deep learning
Source: PLoS Comput Biol. 2021 May 10;17(5):e1008982. doi: 10.1371/journal.pcbi.1008982 (PMC8136849; doi:10.1371/journal.pcbi.1008982)
Supplement: Attachment — Submitted filename: Review of Karollus.docx [file pcbi.1008982.s026.docx]

Review of Karollus, Avsec, Gangeur.

The authors seek to improve on the Optimus algorithm developed by George Seelig’s lab for predicting functional characteristics and repercussions of 5’ untranslated region variants. These earlier algorithms have length limitations, and as the authors describe, the majority of human 5’UTRs are longer than the 50 to 100 nt cutoff. Additionally, positional weight in this algorithm was a concern. By pooling by reading frame contexts, the authors have addressed this concern, as well as improved the ability to predict on longer sequences, more characteristic of human 5’UTRs. Such a tool would be useful in the early detection of de novo variants that could lead to disease. The authors have incorporated this tool into the Kipio API for use with standard variant call format (VCF).

**Section Summaries and Notes**

**Section 1**: The authors describe the frame pooling approach and explain its importance in not generating positional weights when training models. Three models are generated, one trained on the 50 nt dataset, one on the 25-100 variable nt dataset, and a combination of the two.

**Section 2**: The FramePool50 model Is tested on MPRA data and is shown to better predict than Optimus50 for sequences it was not trained on. It is not surprising that a model that was not trained on the experimental data performed poorly (Optimus50), but still, it is impressive that the authors’ model performed well on a dataset it was not trained on.

Note, typo on line 209, the truncated data tests are said to be shown in Fig S2, but this data is shown in Fig S1. Also, apologies if I missed it, but how does the Optimus100 model perform compared to the Framepool100 model when trained and tested on the variable data? I would assume they are similar. How about the combined model? Some additional supplemental figures would be nice here.

**Section 3**: One of the FramePool models accurately predicts strength of effects of upstream translation initiation sites (mutating AGG to AUG). As expected, correlations are negative as strong uTIS’s decrease MRL comparatively more than weak ones. The model also predicts, although not as well, these effects in the context of alternate start codons CUG and GUG.

Why did the authors choose the FramePoolCombined for this task? Is it more comprehensive as it is a combination of the two models?

**Section 4:** The FramePool model predictions on endogenous data are correlated with actual measurements from that data. A slight, but significant, correlation is seen here. Agree with the authors’ point that endogenous data is the result of other contributing sequences and processes than just the 5’UTR here, which is likely the reason the correlation wasn’t stronger. Predictions perform slightly better than the Optimus’ ones for the datasets.

**Section 5:** In silico mutation of nucleotides throughout the 5’UTR. Positions expected to impact highly tend to be more evolutionarily conserved.

**Section 6:** Workflow described, weight matrix used to display effect size and direction for 5’UTR positions along an example gene.

**Major Questions to Address:**

1) I was confused in section 6 around lines 388-390, how to interpret the results of the “shifting”. I understand the concept, that an in frame uTIS will just lengthen the protein, and therefore the MRL may not change much, but am confused about how this is presented by the analysis, or how to interpret results. Please expand upon this.
2) How does the model deal with insertions/deletions?
3) Does framepooling, which will shift the apparent start codon, matter in terms of how far the variant is from this “newly created” start codon?

**Decision:** Recommend publication with minor revisions to address concerns/questions, possibly additional supplemental figures fleshing out initial testing of models in section 2.
